# Auditory Noise Leads to Increased Visual Brain-Computer Interface Performance: A Cross-Modal Study

**DOI:** 10.3389/fnins.2020.590963

**Published:** 2020-12-22

**Authors:** Jun Xie, Guozhi Cao, Guanghua Xu, Peng Fang, Guiling Cui, Yi Xiao, Guanglin Li, Min Li, Tao Xue, Yanjun Zhang, Xingliang Han

**Affiliations:** ^1^School of Mechanical Engineering, Xi’an Jiaotong University, Xi’an, China; ^2^CAS Key Laboratory of Human-Machine Intelligence-Synergy Systems, Shenzhen Institutes of Advanced Technology & Shenzhen Engineering Laboratory of Neural Rehabilitation Technology, Shenzhen, China; ^3^National Key Laboratory of Human Factors Engineering, China Astronauts Research and Training Center, Beijing, China; ^4^State Key Laboratory for Manufacturing Systems Engineering, Xi’an Jiaotong University, Xi’an, China

**Keywords:** brain–computer interface (BCI), steady-state motion visual evoked potential (SSMVEP), auditory noise, cross-modal stochastic resonance, functional connectivity, phase synchronization

## Abstract

Noise has been proven to have a beneficial role in non-linear systems, including the human brain, based on the stochastic resonance (SR) theory. Several studies have been implemented on single-modal SR. Cross-modal SR phenomenon has been confirmed in different human sensory systems. In our study, a cross-modal SR enhanced brain–computer interface (BCI) was proposed by applying auditory noise to visual stimuli. Fast Fourier transform and canonical correlation analysis methods were used to evaluate the influence of noise, results of which indicated that a moderate amount of auditory noise could enhance periodic components in visual responses. Directed transfer function was applied to investigate the functional connectivity patterns, and the flow gain value was used to measure the degree of activation of specific brain regions in the information transmission process. The results of flow gain maps showed that moderate intensity of auditory noise activated the brain area to a greater extent. Further analysis by weighted phase-lag index (wPLI) revealed that the phase synchronization between visual and auditory regions under auditory noise was significantly enhanced. Our study confirms the existence of cross-modal SR between visual and auditory regions and achieves a higher accuracy for recognition, along with shorter time window length. Such findings can be used to improve the performance of visual BCIs to a certain extent.

## Introduction

Brain–computer interface (BCI) is a device which enables users to control a computer or a computer-connected device using brain activity and has shown prospects of broad application (Wolpaw et al., 2000). However, BCI performance has long been limited by the non-linear characteristic of human brain, as well as the weak detectability of electroencephalogram (EEG) signals. Many studies have been conducted and plenty of new paradigms and methods have been presented to solve this problem. Stochastic resonance (SR) theory (Benzi et al., 1981; Collins et al., 1996; Gammaitoni et al., 1998), is one of these methods. Stochastic resonance theory claims that random fluctuation can enhance weak signal input to improve signal transmission and sensitivity to environmental changes in a non-linear system, leading to an improvement in system performance. Such SR effects have been also demonstrated in the neuronal systems, such as the human muscle spindle (Cordo et al., 1996), rat cutaneous mechanoreceptor (Collins et al., 1996), and human tactile sensation perception (Collins et al., 1997).

In the field of BCI, several studies investigating SR have been conducted. Srebro and Malladi (1999) applied two-dimensional spatial temporal noise to traditional visual stimuli which was used to elicit visual evoked potential (VEP). Results indicated that VEP could be enhanced by presenting visual noise. In fact, the power of the second harmonic of the VEP could increase as high as 4.2-fold under conditions of noise, peaking at 30% noise contrast. The power of the fourth VEP harmonic also increased 1.3-fold, peaking at 20% noise contrast. In our previous study, a BCI technology based on pure visual modality SR was proposed (Xie et al., 2012). In the study, subjects were exposed to visual stimuli and visual noise at the same time, which led to an enhancement of nervous system excitability. In 2019, we further evaluated the performance of visual noise imposed on two different BCI paradigms, i.e., motion-reversing simple ring and complex checkerboard (Xie et al., 2019). Additionally, Nakamura et al. (2017) applied auditory noise to auditory steady-state response (ASSR) based BCI and achieved a better performance compared to traditional paradigm, which confirms the existence of a SR effect in the human auditory system.

The studies mentioned above have mainly focused on single-modal SR, that is to say, stimulation and noise belong to the same sensory mode and enter the same sensory channel of the human brain. Besides single-modal SR, cross-modal SR in the human nervous system has also been reviewed (Krauss et al., 2018). Douglass et al. (1993) found that by applying periodic stimulation and environmental noise to the mechanical receptors of crayfish, the periodicity of spike intervals generated by neurons was enhanced. Ross et al. (2006) showed that an appropriate amount of auditory noise is conducive to understanding audiovisual speech and information detection. Kayser et al. (2005) tested changes in the blood oxygen level dependent (BOLD) response of the primate auditory cortex of monkeys to sound stimulation, tactile stimulation, as well as a combination of sound and tactile stimulation, respectively. This study further confirmed that the auditory cortex, including the primary auditory cortex, has integrates auditory and tactile information, and that such integration occurs in early sensory areas. In 2018, Krauss et al. (2018) reviewed these cross-modal enhancement phenomena and speculated that SR in one sensory modality driven by input from another modality may be a general principle, namely multisensory integration causing SR like cross-modal enhancement. However, such cross-modal SR phenomena have not been utilized in the field of BCI yet. Therefore, whether cross-modal SR phenomena can be used to promote BCI performance, like single-modal SR that used in BCI application, remains unclear.

In this study, we applied auditory noise to a steady-state motion visual evoked potential (SSMVEP) (Xie et al., 2012) based BCI paradigm with an oscillating checkerboard stimulation to investigate whether the external auditory noise can lead to an enhancement of SSMVEP responses and improve BCI performance. Gaussian white noise with an intensity of −30, −10, 10, and 30 dBW was selected as auditory noise. The effect of auditory noise on visual responses was verified by both the fast Fourier transform (FFT) spectrum and canonical correlation analysis (CCA) results. We found that BCI performance progressively improved and then decreased with the increment of noise intensities, i.e., a relationship between BCI performance and the moderate increase of noise level. Directed transfer function (DTF) method was applied to investigate the functional connectivity pattern of activated brain regions under different noise levels, which verified the theoretical research, as well as the practical application value, of the proposed BCI paradigm. Furthermore, weighted phase-lag index (wPLI) method was used to analyze the phase synchronization between visual and auditory regions which demonstrated a significant enhancement under moderate auditory noise level. Finally, the analysis on channel combinations and accuracy rate further confirmed the enhancement effect of auditory noise. Our study illustrates the existence of cross-modal SR in the human brain and the enhancement effect of auditory noise, which can be used to enhance visual BCI performance.

## Materials and Methods

### Subjects

Ten subjects from Xi’an Jiaotong University participated in the experiment. Seven were males and three were females (aged 25 ± 3 years old). All subjects had normal or corrected-to-normal hearing and eyesight and had prior experience with SSVEP-BCIs. All subjects had no history of visual or auditory disorders and were not paid for their participation. The experiment was undertaken in accordance with the recommendations of the Declaration of Helsinki. Written informed consent was obtained from each participant, which followed the guidelines approved by the institutional review board of Xi’an Jiaotong University.

### EEG Recordings

**FIGURE 1:**
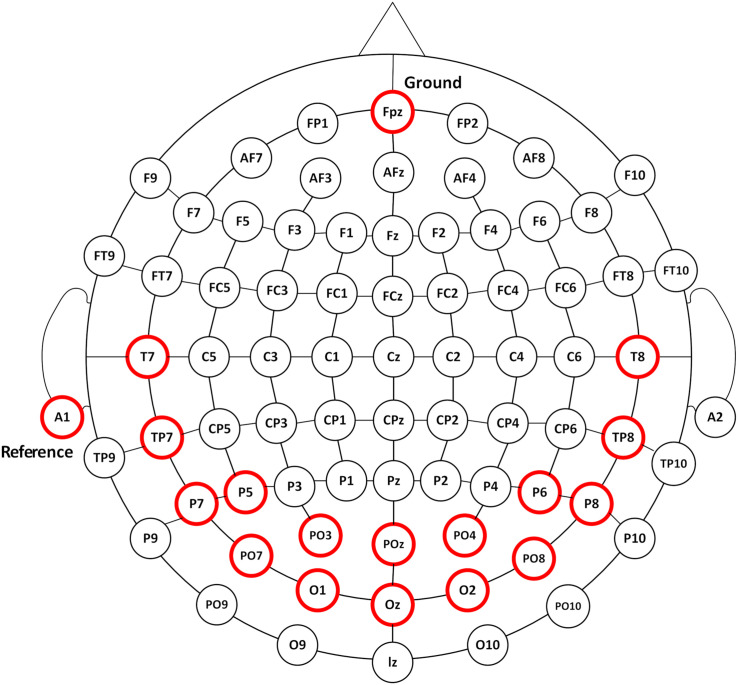
The EEG recording positions. EEG responses were recorded from 16 channels of POz, Oz, PO3, O1, PO4, O2, T7, TP7, T8, TP8, P5, P7, PO7, P6, P8, and PO8 as illustrated in circles in red.

According to the International 10–20 electrodes position system, 16-channel EEG signals were recorded from the occipital, parietal, and temporal areas of POz, Oz, PO3, O1, PO4, O2, T7, TP7, T8, TP8, P5, P7, PO7, P6, P8, and PO8 sites at a sampling rate of 1200 Hz using the g.USBamp system (g.tec, Graz, Austria) (Figure 1). EEG signals were referenced to a unilateral earlobe and grounded over site Fpz. The impedance was kept below 5 K ohm. After application of the analog filter, the EEG signals were filtered between 0.1 and 100 Hz by an 8th-order Butterworth band-pass filter. A notch filter was implemented to remove the power line interference between 48 and 52 Hz with a 4th-order Butterworth band-stop filter. Further analysis was performed in Matlab environment.^1^

### Stimulation Design

**FIGURE 2:**
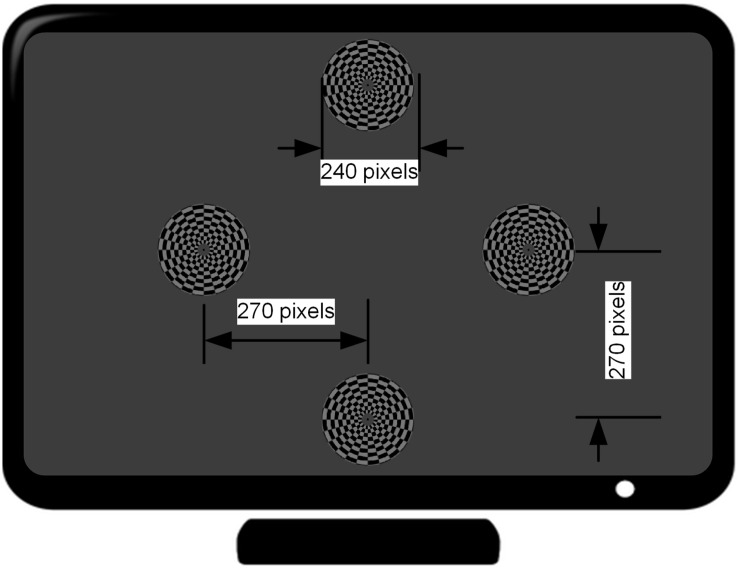
Distribution of four stimulus targets on the computer screen. The distance from the center of the monitor to that of each target is 270 pixels (7.2° visual angle in the case of approximately 80 cm distance between the subject and the monitor).

The motion-reversal visual stimulation, i.e., an oscillating checkerboard, programed by Psychophysics Toolbox^2^ (Brainard, 1997; Pelli, 1997), was introduced as a spatial selective steady-state BCI paradigm. A 27-inch ASUS liquid crystal display (LCD) monitor with a resolution of 1920 × 1080 pixels and a screen refresh rate of 144 Hz was used for the presentation of the visual stimulation. The static image of the oscillating checkerboard was made up of 10 concentric rings (Figure 2). The outer and inner diameters of the motion checkerboard were set to 120 pixels and 12 pixels, respectively. A black spot with radius of 3 pixels was set at the center to keep subjects focused on it during the experiment. Each ring was divided into 24 alternate gray and black blocks. The areas of the bright and dark regions in each ring were equal. The bright color was gray (120, 120, 120) and the dark grids was black (0, 0, 0). The width of each block was set to 10 pixels and subtended a horizontal and vertical visual angle of approximately 4.8° when viewed by the subjects from a fixed distance of approximately 80 cm, in accordance with prior studies which have shown that a stimulation size over 3.8° would saturate brain responses (Ng et al., 2012). The expansion - contraction of the checkerboard constitutes the motion process modulated by a sinusoid function. When the phase of the sinusoidal function shifts from 0 to π, the motion ring contracts with an amplitude of 10 pixels and then expands as the phase shifts from π to 0. Therefore, the direction of motion changes twice in one cycle. This motion direction changing rate is defined as motion-reversal frequency, which is two times the cycle frequency. Since SSMVEP mainly comes from brain activities which are triggered by directional changes, we adopted this motion-reversal frequency as the fundamental frequency of visual stimulation.

Four stimuli were arranged in the corners of a rhombus layout. The distance from each stimulus target to that of the center of monitor is at a 7.2° visual angle (i.e., 270 pixels). Each stimulus target had distinct motion-reversal frequencies. According to a previous report, the low (4–13 Hz), medium (13–30 Hz) and high frequency range (>30 Hz) are the three main frequency ranges to elicit an SSVEP (Regan, 1989). In general, the low frequency range could elicit larger amplitude SSVEP responses than the medium and high frequency ranges. In this study, the frequencies of 7, 9, 11, and 13 Hz were assigned to the left, right, upper, and lower stimulus target, respectively. The four stimuli were simultaneously presented to subjects and the distance between each subject and LCD monitor was set to approximately 80 cm at eye level (Wu and Lakany, 2013). When the subject gazed at the stimulation, auditory noise (i.e., Gauss white noise) was played in both ears of the subject. Due to our previous test, the maximal auditory noise level our subjects can accept is around 30 dBW, and −30 dBW is barely audible. After determining the maximal and minimal level, auditory noise level was graded by equal division of the noise range into four levels by noise power of −30, −10, 10, and 30 dBW, using 1 watt as a baseline. In addition, an experiment was conducted without auditory noise, which constituted the control group. For power calculations, it is assumed that there is a load of 1 Ohm and measure for the output is in Volts. Noise was generated and played using Matlab and presented through a pair of kernel earphones (Sennheiser IE 80s, Germany).

**FIGURE 3:**
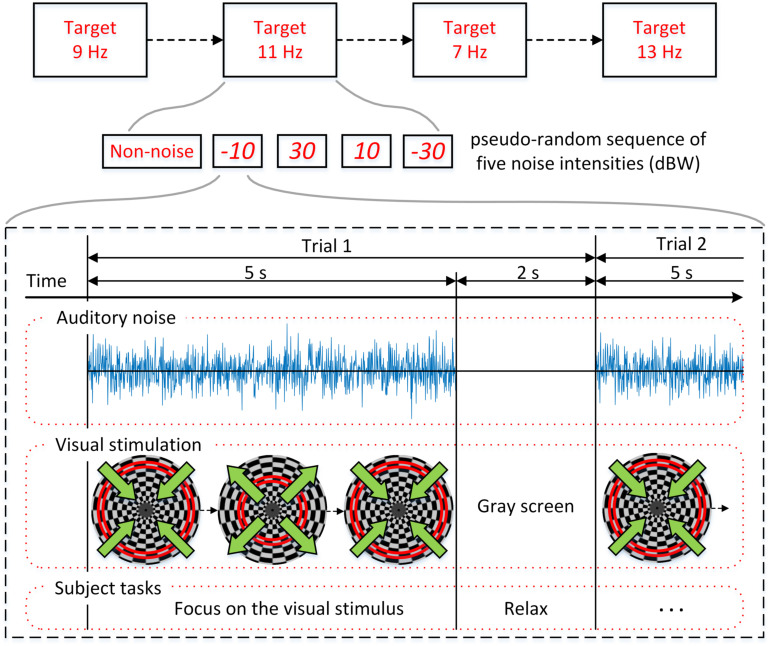
Experimental procedure. Four experiments corresponding to the target stimuli frequencies of 7, 9, 11, and 13 Hz were conducted. Each experiment is consisted of five pseudo-random sequences with different noise intensities.

**FIGURE 4:**
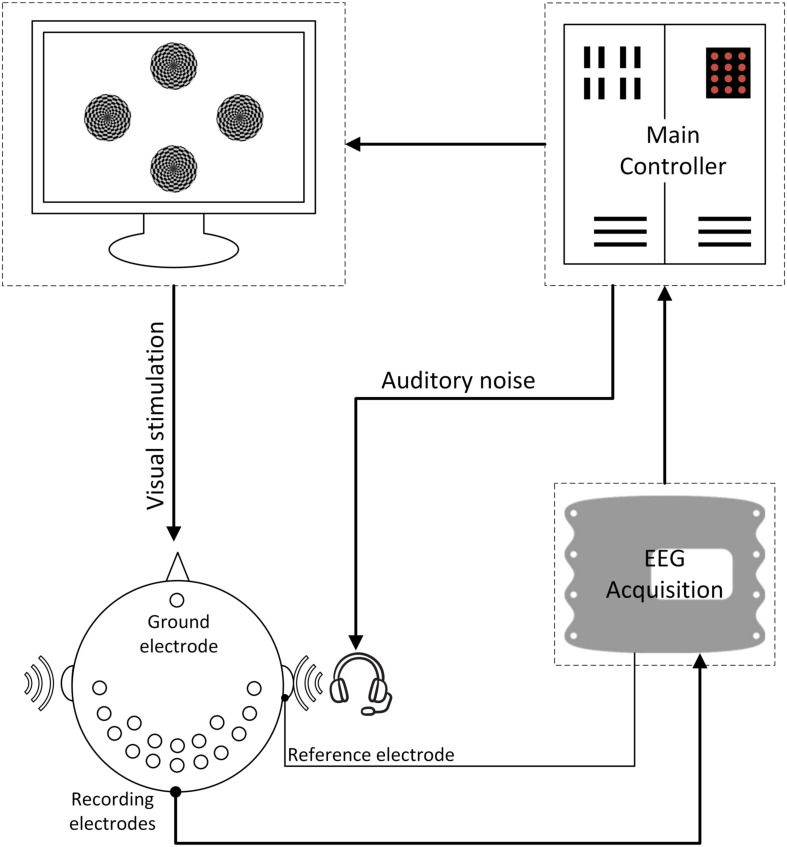
The overall BCI system diagram. During the experiment, the subjects were instructed to only pay attention to one of the stimuli at each single run; meanwhile auditory noise was presented in both ears. EEG signals were recorded in both occipital and temporal brain areas.

The experimental procedure is shown in Figure 3 and the overall BCI system setup is depicted in Figure 4. For each subject, four experiments were conducted for oscillating checkerboard SSMVEP BCI, which corresponded to the target stimuli frequencies of 7, 9, 11, and 13 Hz, respectively. Each experiment contained five runs, which consisted of five pseudo-random sequences of all four auditory noise intensities as well as the non-noise condition. For different target frequencies and different noise levels, the sequences were performed randomly to avoid adaptation and habituation of long-term stimulation that could potentially affect assessment of SR effect (Bergholz et al., 2008). Each run consisted of 20 trials, with each trial lasting 5 s. Between two trials there was a 2-s inter-trial interval (ITI). Additionally, after every two runs, there was a break of 2 min. The whole experiment for each subject lasted approximately 50 min. During each trial, there were four stimuli that were simultaneously presented. The subjects were instructed to only pay attention to one stimulus designated by the operator at each single run; meanwhile auditory noise was presented in both ears. The stimulus target and noise intensity remain unchanged in each single run. During the experiment, the subjects were asked to sit on an armchair in a dim and quiet room. They were not allowed to move their bodies during the experiment and were asked to fixate on the center of screen during the ITI periods.

### DTF and Flow Gain

Directed transfer function, a method that is based on multivariable autoregressive model (MVAR), was used to estimate the brain functional connectivity driven by SSMVEP responses under different noise levels. The EEG data *X* can be described in the following form:

(1) $$\begin{array}{c} X={\left[x_{1}\,\left(t\right),x_{2}\,\left(t\right),\ldots \,x_{n}\,\left(t\right)\right]}^{T} \end{array}$$

where *t* refers to time and *n* refers to the number of channels. Through the use of MVAR, EEG data set *X* can be expressed as the following autoregressive process (Bartels and Zeki, 2004):

(2) $$\begin{array}{c} \sum_{k=0}^{p}A\,\left(k\right)\,X\,\left(t-k\right)=U\,\left(t\right)\;A\,\left(0\right)=1 \end{array}$$

where *p* is the model order chosen with the Akaike information criteria (AIC; Kamiński et al., 2001), i.e., a widely used criteria for AR model order determination. *A*(*k*) represents the *N* by *N* matrix of model coefficients, and *U*(*t*) is a white noise process with zero mean and non-singular covariance matrix.

In order to investigate the spectral features of the examined process, Eq 2 is transformed to the frequency domain:

(3) $$\begin{array}{c} A\,\left(f\right)\,X\,\left(f\right)=U\,\left(f\right) \end{array}$$

where

(4) $$\begin{array}{c} A\,\left(f\right)=\sum_{k=0}^{p}A\,\left(k\right)\,e^{-j\,2\,\pi \,f\,\Delta \,t\,k} \end{array}$$

Hence, Eq 3 can be rewritten as

(5) $$\begin{array}{c} X\,\left(f\right)=A\,{\left(f\right)}^{-1}\,U\,\left(f\right)=H\,\left(f\right)\,U\,\left(f\right) \end{array}$$

*H*(*f*) is the transfer matrix of the system, in which the element *H*_*ij*_ represents a connection between the *j*th input and the *i*th output of the system (Bassett and Bullmore, 2006). Using these definitions, the causal influence of the cortical waveform estimated in the *j*th channel on that estimated in the *i*th channel, i.e., the DTF *H*_*ij*_, can be defined as (Kaminski and Blinowska, 1991):

(6) $$\begin{array}{c} \theta_{i\,j}^{2}\,\left(f\right)={\left|H_{i\,j}\,\left(f\right)\right|}^{2} \end{array}$$

The normalization of DTF matrix constructed above is as follows (He et al., 2011):

(7) $$\begin{array}{c} \gamma_{i\,j}^{2}\,\left(f\right)=\frac{{\left|H_{i\,j}\,\left(f\right)\right|}^{2}}{\sum_{m=1}^{N}{\left|H_{i\,m}\,\left(f\right)\right|}^{2}} \end{array}$$

γ_*ij*_(*f*) represents the ratio of influence of the cortical waveform estimated in the *j*th channel on the cortical waveform estimated on the *i*th channel, with respect to the influence of all estimated cortical waveforms. Normalized DTF values are in the interval [0,1] when the normalization condition of

(8) $$\begin{array}{c} \sum_{n=1}^{N}\gamma_{i\,n}^{2}\,\left(f\right)=1 \end{array}$$

is applied.

The inflow and outflow of the information transmission process in the brain can be defined as $\sum_{j=1}^{N}\gamma_{m\,j}^{2}$ and $\sum_{i=1}^{N}\gamma_{i\,m}^{2}$, respectively. The inflow indicates the magnitude of all the incoming links from the other channels. This information depicts each channel as the target of functional connections from the other channels. On the contrary, the outflow, depicting each channel as the source, indicates the magnitude of the considered channel linking out toward the others (Yan and Gao, 2011).

Hence, flow gain value was defined as the ratio of outflow to inflow. For channel *m*:

(9) $$\begin{array}{c} \rho_{m}=\frac{\sum_{i=1}^{N}\gamma_{i\,m}^{2}}{\sum_{j=1}^{N}\gamma_{m\,j}^{2}} \end{array}$$

The value of ρ_*m*_ represents the contribution that channel *m* plays during information transmission process, and a higher value represents more contribution of information output during the transmission process.

### CCA Method

Canonical correlation analysis is one of the most commonly used algorithms to measure the maximum correlation between two sets of multidimensional variables in multi-channel SSVEP-based BCIs (Lin et al., 2006; Xie et al., 2012). In this case, we used CCA algorithm to compare actual EEG signals with reference signals to identify their correlation coefficients. The reference signals are defined as a set of cosine and sine signals with the fundamental frequency and harmonics as follows:

(10) $$\begin{array}{c} Y_{i}=\left(\begin{array}{c} \cos\left(2\,\pi \cdot f_{i}\cdot t\right)\\ \sin\left(2\,\pi \cdot f_{i}\cdot t\right)\\ \cdot \\ \cdot \\ \cdot \\ \cos\left(2\,\pi \cdot H\,f_{i}\cdot t\right)\\ \sin\left(2\,\pi \cdot H\,f_{i}\cdot t\right) \end{array}\right),t=\frac{1}{F_{s}},\ldots ,\frac{S}{F_{s}} \end{array}$$

where *F*_*s*_ refers to the sampling rate, *H* is the number of harmonics, *f*_*i*_ is the stimulus frequency, *t* is the discrete time series of predefined time-window length, and *S* is sampling numbers. The set of EEG signals are defined as follows:

(11) $$\begin{array}{c} X=\left(\begin{array}{c} \begin{array}{c} x_{1}\,\left(t\right)\\ \begin{array}{c} \cdot \\ \cdot \\ \cdot \end{array} \end{array}\\ x_{n}\,\left(t\right) \end{array}\right),t=\frac{1}{F_{s}},\ldots ,\frac{S}{F_{s}} \end{array}$$

where *x* refers to EEG signals recorded from each single channel and *n* refers to the channel number.

Given the multivariable matrices of *X* and *Y*_*i*_, CCA first projects them into one dimension by the two weight vectors *W*_*x*_ and *W*_*y_i*_, and then calculates their correlation coefficients in one-dimensional space. CCA seeks the weight vectors *W*_*x*_ and *W*_*y_i*_ to maximize their linear correlation ρ_*x,y_i*_:

(12) $$\begin{array}{c} x=X^{T}\,W_{x}\\ y_{i}=Y_{i}^{T}\,W_{y_{i}}\\ \rho_{x,y_{i}}=\frac{c\,o\,v\,\left(x,y_{i}\right)}{\sqrt{D\,\left(x\right)}\,\sqrt{D\,\left(y_{i}\right)}} \end{array}$$

where ρ_*x*,*y*_*i*__ indicates the canonical correlation between *X* and *Y*_*i*_, and the stimulus frequency *f*_*i*_ (*i* = 1, …, *K*) can be recognized based on maximum ofρ_*f*_*i*__.

With the corresponding correlation coefficient ρ_*f_i*_, CCA can be performed on each stimulus frequency *f*_*i*_ (*i* = 1, …, *K*) separately. Then the target *f*_*target*_ can be recognized as:

(13) $$\begin{array}{c} f_{t\,a\,r\,g\,e\,t}=\underset{i=1,,K}{\text{max}}\rho_{f_{i}} \end{array}$$

Here, the stimulus frequency *f*_*i*_ (*i* = 1, …,4) is set to the frequency of each oscillating checkerboard, the number of *C* channels was set to 16, and the harmonics of *H* was set to 1.

### wPLI Method

The wPLI method (Vinck et al., 2011) analyzes phase synchronization between two time series *x*(*t*) and *y*(*t*). Weighted phase-lag index uses only the imaginary component of the cross-spectrum and is immune to both volume conductor effect and measurement noise. At the same time, wPLI exhibits increased sensitivity to phase interactions between signals (Vindiola et al., 2014). The instantaneous phase lag and magnitude is acquired through cross power density spectrums:

(14) $$C\,\left(f\right)=\int_{-\infty }^{+\infty }X\,\left(f\right)\cdot Y\,\left(t-f\right)\cdot d\,t$$

where *X*(*f*) and *Y*(*f*) are finite Fourier transform of signal *x*(*t*) and *y*(*t*).

Then wPLI index is calculated as follow:

(15) $$w\,P\,L\,I=\frac{\left|E\,\left\{\left\{C\right\}\right\}\right|}{E\,\left\{\left|\left\{C\right\}\right|\right\}}$$

where {*C*} is the imaginary component of the cross-spectrum *C*(*f*).

The value of wPLI index is limited between 0 and 1, with a higher value representing stronger phase synchronization.

### Statistical Analyses

The values of each individual subject across the non-noise and auditory noise integrated BCI conditions were analyzed using the one-way analysis of variance (ANOVA) statistic. The level of statistical significance was set to *p* < 0.05. Bonferroni correction was employed for multiple comparisons. The results were expressed as mean ± standard deviation (SD).

## Results

### The Influence of Auditory Noise on Visual Responses

**FIGURE 5:**
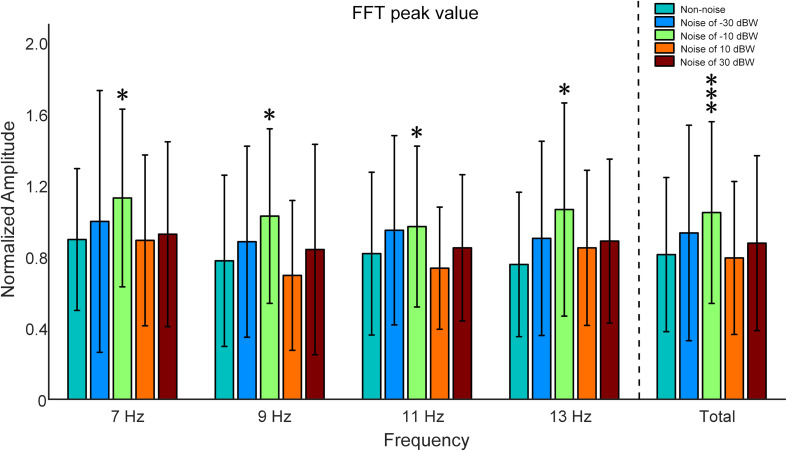
Comparison of normalized SSMVEP spectral amplitudes with a change of noise levels across subjects. All statistics were assessed using one-way ANOVA, *p < 0.05 represent significance among non-noise and auditory noise integrated BCI tasks, ***p < 0.001 among non-noise and auditory noise integrated BCI tasks.

In order to examine the influence of auditory noise on visual responses, EEG responses acquired from the temporal-parietal but not the occipital area were used to analyze the response amplitude changes under different auditory noise levels. Fast Fourier transform was performed on the EEG data obtained from T7, P7, TP7, T8, P8, and TP8 channels in this study. Inter-subject normalization was attained by dividing amplitude estimates by the average computed from all amplitude values of both non-noise and auditory noise integrated conditions, but separately for each subject (Xie et al., 2017). There is a resonance between normalized FFT values and the intensities of auditory noise, i.e., moderate auditory noise enhanced the FFT value while too much noise weakened it (Figure 5). For target frequency of 7 Hz, normalized SSMVEP spectral amplitudes significantly increased by 25.97% at auditory noise level of −10 dBW, when compared to the non-noise condition and other noise intensities (−10 dBW: 1.1277 ± 0.4977, non-noise condition: 0.8952 ± 0.3974, one-way ANOVA: *F* = 2.4005, *p* = 0.0498). For target frequency of 9 Hz, normalized SSMVEP spectral amplitudes significantly increased by 32.30% at noise level of −10 dBW in comparison to the non-noise condition and other noise intensities (−10 dBW: 1.0265 ± 0.4890, non-noise condition: 0.7759 ± 0.4796, *F* = 2.4210, *p* = 0.0498). For target frequency of 11 Hz, normalized SSMVEP spectral amplitudes significantly increased by 18.58% at noise level of −10 dBW in comparison to the non-noise condition and other noise intensities (−10 dBW: 0.9676 ± 0.4507, non-noise condition: 0.8160 ± 0.4561, *F* = 2.8344, *p* = 0.0248). For target frequency of 13 Hz, normalized SSMVEP spectral amplitudes also significantly increased by 40.75% at noise level of −10 dBW compared with non-noise condition and other noise intensities (−10 dBW: 1.0632 ± 0.5971, non-noise condition: 0.7554 ± 0.4046, *F* = 2.5683, *p* = 0.0387). The average value for all four frequencies at noise level of −10 dBW is 1.0510 (SD = 0.5090), which is 28.16% higher than that of non-noise condition and other noise intensities (*F* = 9.0782, *p* < 0.001).

### Auditory Noise Promoted Visual BCI Recognition Accuracy

**FIGURE 6:**
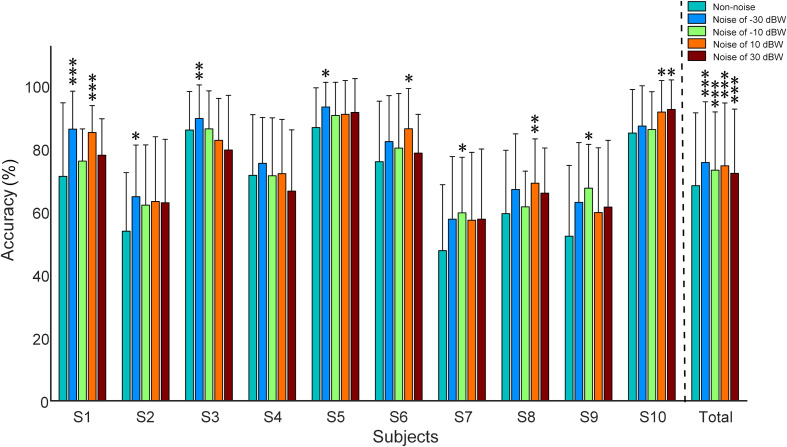
Recognition accuracy rates under different auditory noise levels when subjects gazed at a target stimulus of 7 Hz. All statistics were assessed by one-way ANOVA, *p < 0.05 represent significance among non-noise and auditory noise integrated BCI tasks, **p < 0.01 among non-noise and auditory noise integrated BCI tasks, ***p < 0.001 among non-noise and auditory noise integrated BCI tasks.

Figure 6 shows the recognition accuracy of all subjects under visual stimulus frequencies of 7, 9, 11, and 13 Hz. Recognition accuracy, obtained using the CCA recognition algorithm, is defined as the number of correct selections divided by total number of trials. All 16 channels that involve visual and auditory brain areas were selected for analysis. Considering the fact that long time window would possibly lead to high accuracy values even in multi-choice SSVEP BCI (i.e., the ceiling effect), which would make it difficult to inspect the impact of auditory noise on visual BCI performance, the 5-s single-trial data was truncated into 0.25 s and was consequently analyzed. Consistent with the phenomena observed in the normalized SSMVEP spectra of visual responses, a resonance is reached between the BCI accuracy and the noise intensity (Figure 6). Additionally, for Subject S1, S2, S3, S5, S6, S7, S8, S9, and S10, moderate auditory noise at the resonance points significantly improved BCI accuracies (*F* = 6.3667, *p* < 0.001 for Subject S1; *F* = 2.6921, *p* = 0.0316 for Subject S2; *F* = 4.2652, *p* = 0.0023 for Subject S3; *F* = 2.6689, *p* = 0.0328 for Subject S5; *F* = 2.8481, *p* = 0.0249 for Subject S6; *F* = 3.2148, *p* = 0.0132 for Subject S7; *F* = 3.8410, *p* = 0.0046 for Subject S8; *F* = 3.2224, *p* = 0.0137 for Subject S9; *F* = 2.9871, *p* = 0.0204 for Subject S10). However, it was not significant for Subject S4 (*F* = 2.3666, *p* = 0.0524). For grand accuracies across subjects, the accuracy rates of all auditory noise levels (i.e., −30, −10, 10 dBW as well as 10 dBW) were significantly higher than that of non-noise condition (*F* = 9.8923, *p* < 0.001). Such results indicate that there exist optimal noise intensities that can improve the BCI performance using cross-modal SR effect.

### Auditory Noise Optimized the Trade-off Between Time-Window Length and Performance in Accuracy

**FIGURE 7:**
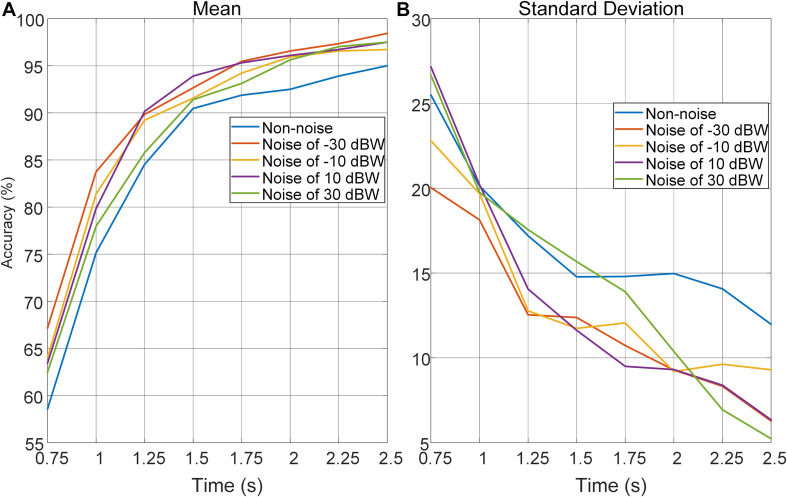
Average recognition accuracies and corresponding standard deviations obtained across all target frequencies, noise levels and subjects by CCA method with different time-window lengths. **(A)** Average recognition accuracies with different time-window lengths. **(B)** Standard deviations of accuracies with different time-window lengths.

In order to further investigate the effect of auditory noise on the trade-off between time-window length, performance in accuracy, and stability of auditory noise integrated BCI paradigm, EEG data was truncated to different time-window lengths within the 5-s single-trial duration. As the time window increased gradually in steps of 0.25 s from 0.75 to 2.5 s, the changes in mean accuracy rates and corresponding standard deviations obtained across all target frequencies and all subjects, using the CCA method, are shown in Figure 7. Brain–computer interface accuracies showed sustainable improvement with increasing time-window lengths for both non-noise and auditory noise integrated BCI tasks. Overall, the accuracies of auditory noise integrated tasks under noise levels of −30, 10, and 30 dBW were higher than that of non-noise task as time-window length increases from 1.25 to 5 s (*F* = 6.5139, *p* < 0.001). For auditory noise level of 10 dBW, the average accuracy exceeded 90% for a time window of 1.25 s, and 95% for a time window of 1.5 s, indicating that the auditory noise integrated paradigm can achieve a high performance in a short time window (Figure 7A). Comparisons of standard deviations between non-noise and auditory noise integrated paradigms are depicted in Figure 7B. Compared to the non-noise condition, the standard deviations of accuracies of auditory noise integrated tasks under noise levels of −30, 10, and 30 dBW drop sharply as time-window length increases from 1.25 to 5 s (*F* = 5.6619, *p* < 0.001). In particular, for a time-window length of 1.5–2.5 s, the standard deviations of accuracies of noise level 10 dBW were almost one-half to one-third of the standard deviations under non-noise condition. The comparatively lower standard deviations related to the auditory noise integrated tasks suggest that auditory noise integrated BCI can achieve a more stable performance in accuracy compared to the ordinary non-noise paradigm. Taken together, the optimal auditory noise level of 10 dBW concurrently achieved both higher accuracy and lower standard deviations. This indicates that when compared with non-noise condition, it took less time to achieve a higher recognition accuracy and more stable BCI performance when adding moderate auditory noise to subjects in visual BCI application. Thus, the trade-off between time-window length and performance in accuracy, a common problem in BCI, can be optimized through the cross-modal SR effect.

### Illustration of the Functional Connectivity Under Different Auditory Noise Levels via Flow Gain Maps

**FIGURE 8:**
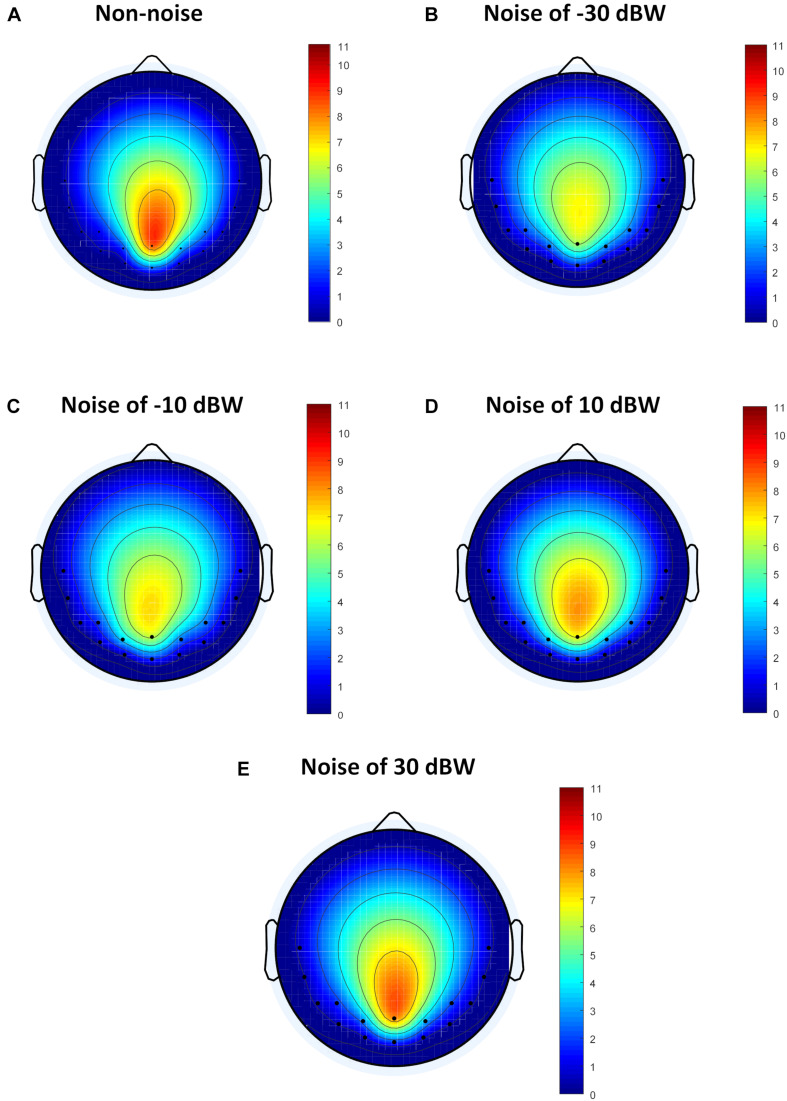
The average flow gain maps under different auditory noise levels. **(A)** Non-noise condition. **(B)** Noise level of −30 dBW condition. **(C)** Noise level of −10 dBW condition. **(D)** Noise level of 10 dBW condition. **(E)** Noise level of 30 dBW condition.

Although the method of analysis in frequency domain such as FFT can analyze the influence of auditory noise on visual BCI responses, the functional connection between different regions caused by auditory noise in the brain, such as connectivity between occipital lobe and temporal lobe, remains unclear. For different auditory noise intensities, the eConnectome toolbox (He et al., 2011), based on the DTF, was applied to analyze the direct interconnections of different brain regions. Flow gain value was defined as the ratio of outflow to inflow of information in a certain channel in order to measure the contribution a channel plays in the information transmission process. As a ratio of outflow to inflow, flow gain value integrates input and output information simultaneously, so that the results shown by flow gain are more direct and clearer. A higher flow gain value indicates that the region makes more contributions to other regions. The topographic distributions of the flow gain values form the corresponding flow gain map. Figure 8 shows the averaged flow gain maps of SSMVEP responses under different auditory noise intensities. The results were an average of all ten subjects and all four stimulus frequencies. As expected, it can be seen on the flow gain maps that under the non-noise condition, the EEG responses were mainly involved in the occipital region. Then with increments of auditory noise levels, the EEG responses started to gradually expand outward from the occipital region to bilateral temporal cortices, which represented a wider region of activation in the brain. Additionally, when the auditory noise level reached to 30 dBW, the connectivity between occipital lobe and temporal lobe lessened. In this study, the flow gain values between temporal region (T7 and T8 sites) and occipital region (O1 and O2 sites) were compared. For non-noise condition, statistical results showed that the flow gain values of temporal region are comparable with that of occipital region with no statistical significance (*F* = 0.0273, *p* = 0.8694). With the increase of the noise intensity, the flow gain values of temporal region are significantly higher than that of occipital region under noise level of −30 and −10 dBW (*F* = 4.3677, *p* = 0.0407 for −30 dBW; *F* = 4.1331, *p* = 0.0463 for −10 dBW). When further increasing the noise level, no statistical significant flow gain difference can be found between temporal and occipital regions (*F* = 2.7200, *p* = 0.1042 for 10 dBW; *F* = 2.1168, *p* = 0.1507 for 30 dBW). From the flow gain maps and corresponding statistical analysis, we can conclude that moderate noise can activate wider area of brain, while too much inhibits it. This result qualitatively evaluated the functional connectivity between visual and auditory areas of the brain under different auditory noise levels.

### The Phase Synchronization Between Temporal Region and Occipital Region

**FIGURE 9:**
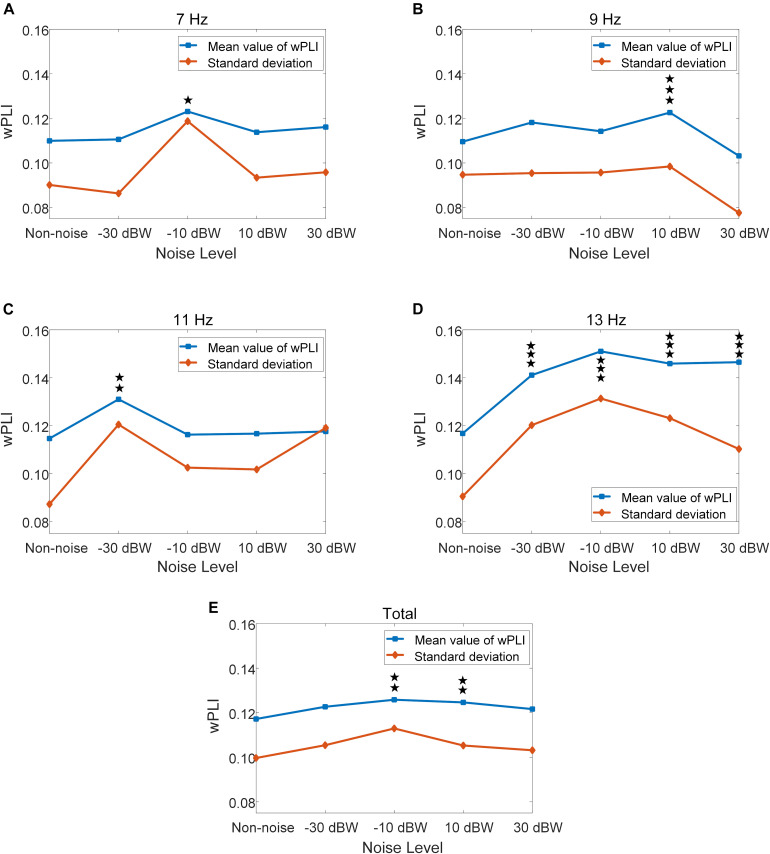
Mean and standard deviation of wPLI values under different auditory noise levels, *p < 0.05 represent significance among non-noise and auditory noise integrated BCI tasks, **p < 0.01 among non-noise and auditory noise integrated BCI tasks, ***p < 0.001 among non-noise and auditory noise integrated BCI tasks. **(A)** 7 Hz condition. **(B)** 9 Hz condition. **(C)** 11 Hz condition. **(D)** 13 Hz condition. **(E)** Total of four frequency condition.

For further quantitative evaluation of neural interactions between the temporal and occipital region under different noise levels, we implemented a more sophisticated analysis using wPLI to quantify the phase synchronization between these two regions. The wPLI between T7-O1 sites, as well as T8-O2 sites, of all ten subjects are calculated. The values of wPLI across all ten subjects for stimulus frequencies of 7, 9, 11, 13 Hz exhibited an enhancement by moderately increasing the noise intensity (Figure 9). Statistical analysis indicated that, for the total results of the four frequencies, wPLIs under −10 and 10 dBW are significantly higher than that under the non-noise condition (*F* = 4.3340, *p* = 0.0017) (Figure 9E). Additionally, wPLI values increased from 0.1172 ± 0.0997 (range: 0.0175–0.2169) under non-noise condition to a maximum of 0.1258 ± 0.1130 (range: 0.0128–0.2388) under noise level of −10 BW condition. Specifically, at stimulus frequency of 7 Hz, wPLIs significantly increased by 12.0% from 0.1099 ± 0.0901 (range: 0.0198–0.2000) under non-noise condition to a maximum of 0.1231 ± 0.1187 (range: 0.0044–0.2418) under noise level of −10 dBW (*F* = 3.2071, *p* = 0.0122) (Figure 9A). At stimulus frequency of 9 Hz, wPLIs significantly increased by 11.9% from 0.1096 ± 0.0947 (range: 0.0149–0.2043) under non-noise condition to a maximum of 0.1226 ± 0.0984 (range: 0.0242–0.2210) under noise level of 10 dBW (*F* = 5.9517, *p* < 0.001) (Figure 9B). At 11 Hz, wPLIs significantly increased by 14.2% from 0.1147 ± 0.0873 (range: 0.0274–0.2020) under non-noise condition to a maximum of 0.1310 ± 0.1205 (range: 0.0105–0.2515) under noise level of −30 dBW (*F* = 3.4980, *p* = 0.0074) (Figure 9C). Lastly, at 13 Hz, wPLIs significantly increased by 29.3% from 0.1168 ± 0.0905 (range: 0.0263–0.2073) under non-noise condition to a maximum of 0.1510 ± 0.1313 (range: 0.0197–0.2823) under noise level of −10 dBW (*F* = 14.85, *p* < 0.001) (Figure 9D). All these results indicate that the neural interaction between visual and auditory brain areas were quantitatively enhanced by the cross-modal SR effect with the combination of visual stimulation and auditory noise.

### Optimal Noise Activates More Channels

**FIGURE 10:**
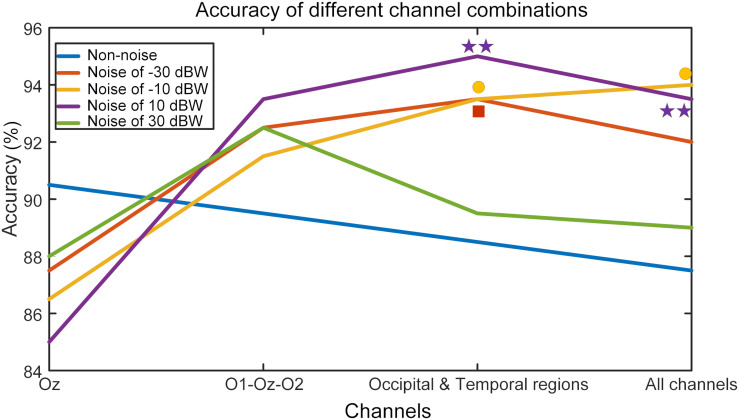
BCI accuracy rates in different channel combination conditions. The mark above each curve indicates that the value of corresponding point is significantly higher than that of the single Oz channel, *p < 0.05 represent significance among different channel combinations, **p < 0.01 among different channel combinations.

In order to study the resonance effect of auditory noise on activation of different EEG sites, we calculated BCI accuracy in different EEG recording channel combinations (Figure 10). EEG channels were divided into four different combinations. The first was the single Oz-channel condition, the second was the O1-Oz-O2 three-channel combination, the third encompassed the channels from occipital-temporal region (Oz, O1, O2, PO3, PO4, POz, T7, P7, TP7, T8, P8, TP8) condition and fourth was the all 16-channel combination. For the non-noise task, the accuracy rate decreased as more channels became involved but without any statistical significance. However, for an auditory noise integrated task, the results were surprisingly different. Under the noise level of −30 dBW, the accuracy rate at a channel combination condition of occipital-temporal region was higher compared to the single Oz condition (*F* = 3.1018, *p* = 0.0301). Additionally, for noise level of −10 and 10 dBW conditions, the accuracy rates at channel combination of occipital-temporal region, as well as all 16-channel combination, were significantly higher compared to that of single Oz condition (*F* = 3.7910, *p* = 0.0127 and *F* = 4.6986, *p* = 0.0040, respectively). However, for the 30 dBW noise level condition, while the trend was similar to noise level of −30 dBW condition, no statistical significance was found (*F* = 1.9126, *p* = 0.1324). These results indicate a small amount of noise can enhance occipital EEG responses, demonstrated by the increased accuracy in the channel combination of occipital-temporal region on noise level of −30 dBW. With further increments of auditory noise intensity such as −10 and 10 dBW, such effect spread to a wider region, including the temporal region, which is demonstrated by the increased accuracy in occipital-temporal channel combination on noise level of 10 dBW. Furthermore, when the noise level was too high, e.g., at 30 dBW, such enhancement effect would attenuate and the accuracy rate decreased.

## Discussion

While noise can often be a harmful component, for dynamic and non-linear system such as human brain, noise can help improve system performance, as explained by the SR theory. Several studies that have explored both single-modality SR and cross-modality SR phenomena, have proven this theory (Srebro and Malladi, 1999; Xie et al., 2012; Nakamura et al., 2017; Krauss et al., 2018). In this study, we applied Gaussian white auditory noise with intensities of −30, −10, 10, and 30 dBW during SSMVEP-BCI experiment to explore the cross-modal SR effect between human visual and auditory modality.

In this study, FFT analysis revealed that, when compared to non-noise conditions, additional auditory noise did raise peak FFT value at a target frequency, proving that SSMVEP response could be enhanced using auditory noise. The BCI accuracy rate obtained using the CCA method further revealed this phenomenon. As noise intensity moderately increases, the correct rate of BCI recognition performance first increased and then decreased. This finding is consistent with previous studies in single-modality BCIs, which demonstrate that moderate noise can enhance BCI performance (Srebro and Malladi, 1999; Xie et al., 2014; Nakamura et al., 2017). In this study, we showed that the proposed cross-modal BCI leads to a similar conclusion. However, it should be noted that the participant variability has a distinct impact on the experimental results. The optimal noise level varies with different subjects and stimulus frequencies. For some subjects, certain noise can cause a sudden drop on peak FFT value, such as Subject S2 at noise level of −30 dBW, and Subject S4 and S5 at noise level of 10 dBW. This may be due to high variability of sensory thresholds and internal noise sources of humans, leading to different sensitivities of neurons in the visual cortex (Srebro and Malladi, 1999). To reduce the impact of participant variability, there will be a larger participant sample in our future work.

From the perspective of time window, we found that it took less time to achieve a higher recognition accuracy when we added moderate auditory noise. In other words, under additional auditory noise, our brain tends to be more sensitive to steady-state visual stimuli, and the response time of the BCI system is shortened. This is especially true in the time-window length of 0.75–1.25 s, in which optimal auditory noise benefits much higher accuracy rate compared to non-noise condition. Interestingly, such phenomenon is in accordance with Harper’s finding (Harper, 1979) in 1979, which is much earlier than the first time the SR theory was defined (Benzi et al., 1981). Here, the accuracy rate was obtained through CCA method, which is one of the most commonly used algorithms in SSVEP-BCI recognition. Furthermore, we believe that, with more powerful algorithms, better performance can be achieved in future work. Since accuracy rate of the proposed BCI paradigm has to be improved to a larger extent, and the response speed can also be accelerated via the usage of the proposed cross-modal modality, this proposed BCI paradigm can help potentially build high speed SSVEP-BCI systems.

In this analysis, we also drew flow gain maps to further investigate the role that auditory noise plays in the interaction between different brain regions. Under noise levels of −30, −10, and 10 dBW, EEG responses may spread to more brain regions compared to the non-noise condition. However, for the noise level of 30 dBW, this effect may attenuate. Considering the results of the FFT response and accuracy rate obtained by CCA method, such results can be anticipated. For single-modal SR, such as in the pure visual or auditory sensory pathway, SR effect can be explained as additive noise that turns neurons from subthreshold to superthreshold (Xie et al., 2014; Tanaka et al., 2015). However, in the current study, the underlying mechanism is more complicated since auditory noise and visual stimulation belong to two different sensory pathways.

The wPLI results are helpful when it comes to understanding the underlying mechanism. As the noise intensity increases, the wPLI values first increase and then decrease, just as observed in FFT value and BCI accuracy. Although the absolute value is not high, statistical analysis indicates that this conclusion is robust. On one hand, low absolute value indicates that the normal neural interaction between auditory and visual regions is relatively weak. On the other hand, the relationship between wPLI values and auditory noise levels implies that the auditory noise enhances synchronization between temporal and occipital regions, and such enhancement is consistent with enhancement of brain responses and BCI performance, as characterized by the SR effect. At stimulus frequencies of 11 and 13 Hz, the BCI performance elevation under optimal noise condition, compared to the non-noise condition, could be as high as 24 and 43%, respectively. Furthermore, from the analysis of different channel combinations, we can see how such effect changes with increases in noise level. When applied to a noise intensity of −30 dBW, SR effect concentrates on the visual region and combination of other channels even weakens the efficiency of target recognition. At noise level of −10 and 10 dBW tasks, there is no significant accuracy differences between O1-Oz-O2 combination condition and the single Oz-channel condition. Once the auditory region related EEG channels were included, the BCI accuracy rate significantly increased.

Based on these findings, we can extrapolate that cross-modal SR may involve integration of different sensory processing regions. In fact, in sensory processing, cross-modal interactions are quite common and many studies have further confirmed this phenomenon. For example, it has been proven that the dorsal cochlear nucleus, the earliest processing stage in the auditory pathway, receives not only input from the cochlea, but also from the somatosensory system that process tactile information (Ryugo et al., 2003; Shore and Zhou, 2006; Dehmel et al., 2012; Zeng et al., 2012). Furthermore, Huang et al. (2017) found that electro-tactile stimulation applied to the index finger significantly improves speech perception thresholds. As for audio-visual integration, it is well-known that sometimes hearing can be misled by vision input, which is well-known as the McGurk effect (McGurk and MacDonald, 1976). Additionally, Caclin et al. (2011) found that visual perception can be enhanced by auditory stimulation, and even subthreshold visual stimuli may be perceived through spatially converging audio-visual inputs (Bolognini et al., 2005). For these cross-modal improvement phenomena, Krauss et al. (2018) speculated that SR in one sensory modality driven by input from another modality may be a general principle, namely multisensory integration, which would cause SR-like cross-modal enhancement. Our findings in this study support this speculation.

## Conclusion

In this study, we propose an auditory-noise-enhanced visual SSMVEP-BCI paradigm with application of cross-modal SR mechanism. The results indicate that moderate auditory noise can increase BCI recognition accuracy and reduce response time, which provides a novel method to improve BCI performance. The combination of flow gain maps and wPLI values both qualitatively and quantitatively revealed that the existence of auditory noise may spread EEG responses to a wider brain area. Furthermore, this phenomenon could be caused by enhancing neural interaction between auditory and visual pathways via the cross-modal auditory-noise-induced SR mechanism. Such findings reveal the principle of cross-modal SR of the brain and provide a potentially novel approach for designing more effective audiovisual hybrid BCI systems.

## Data Availability Statement

The raw data supporting the conclusions of this article will be made available by the authors, without undue reservation.

## Ethics Statement

The studies involving human participants were reviewed and approved by the institutional review board of Xi’an Jiaotong University. The patients/participants provided their written informed consent to participate in this study.

## Author Contributions

JX conceived the study, participated in the design of the study, and carried out the experiments. GZC carried out the experiments and wrote the manuscript. GHX, GLC, YX, and PF designed the study. TX and YJZ carried out the experiments and collected the data. XLH carried out the statistical data analyses. GLL and ML corrected the language. All authors contributed to the article and approved the submitted version.

## Conflict of Interest

The authors declare that the research was conducted in the absence of any commercial or financial relationships that could be construed as a potential conflict of interest.

## Footnotes

1 http://www.mathworks.com

2 http://psychtoolbox.org/
